# Quantifying benefit-risk preferences for new medicines in rare disease patients and caregivers

**DOI:** 10.1186/s13023-016-0444-9

**Published:** 2016-05-26

**Authors:** T. Morel, S. Aymé, D. Cassiman, S. Simoens, M. Morgan, M. Vandebroek

**Affiliations:** KU Leuven Department of Pharmaceutical and Pharmacological Sciences, Leuven, Belgium; INSERM, US14, Paris, France; Hepatology Department, Department of Metabolic Diseases, University Hospital Leuven, Leuven, Belgium; KU Leuven Department of Pharmaceutical and Pharmacological Sciences, Herestraat 49, 3000, Leuven, Belgium; Institute of Pharmaceutical Science, Faculty of Life Sciences & Medicine, King’s College London, London, UK; KU Leuven Faculty of Economics and Business and Leuven Statistics Research Centre, Leuven, Belgium

**Keywords:** Rare diseases, Patients, Caregivers, Benefit-risk, Preferences, Values, Trade-offs, Risk tolerance, Discrete choice experiment, Qualitative survey, Disability, Patient-centered outcomes, Patient-reported outcomes, Drug development

## Abstract

**Background:**

Rare disease patients and caregivers face uncommon, serious, debilitating conditions often characterised by poor prognosis and limited treatment options. This study aimed to explore what they consider of value when choosing between hypothetical therapeutic options and to quantify both their benefit-risk preferences and the influence of disease context.

**Methods:**

A mixed-methods survey with patients and caregivers was conducted in the United Kingdom across a range of rare diseases. Discrete-choice experiments that compared hypothetical treatment profiles of benefits and risks were used to measure respondent preferences across a set of seven attributes related to health outcomes, safety, and process of care. Bespoke questions on current disease management and the joint use of the 12-item WHODAS 2.0 questionnaire and of two Likert scales capturing self- and proxy-assessed disease-induced threat to life and impairment were implemented to describe disease context. Additionally, qualitative insights on the definitions of value and risk were collected from respondents.

**Results:**

Final study sample included 721 patients and 152 informal caregivers, across 52 rare diseases. When choosing between hypothetical novel treatments for rare diseases, respondents attributed most importance to drug response, risk of serious side effects, and the ability to conduct usual activities while on treatment. In contrast, attributes related to treatment modalities were the least important. Respondents expressed a willingness to accept risks in hopes of finding some benefit, such as a higher chance of drug response or greater health improvement potential. Increasing disease severity, impairment or disability, and the lack of effective therapeutic options were shown to raise significantly the willingness to gain benefit through increased risk.

**Conclusions:**

This is the first study performing a quantitative discrete choice experiment amongst patients and caregivers across 52 rare conditions. It enables a more detailed understanding of the relationship between disease context, treatment attributes and the degree of risk respondents are willing to take to gain a specific degree of benefit. Researchers of novel therapeutics for rare diseases should be encouraged to invest in preference elicitation studies to generate rigorous patient evidence and specific regulatory guidance should be issued to acknowledge their importance and their use in marketing authorisations.

**Electronic supplementary material:**

The online version of this article (doi:10.1186/s13023-016-0444-9) contains supplementary material, which is available to authorized users.

## Background

Regulatory decisions are typically depicted as a balancing act between benefits and risks where both interpretations of hard clinical data and value judgements interplay. Traditionally, regulators have made assumptions, albeit implicit, about what patients actually think and prefer, including their willingness to trade benefits for harms [1, 2]. Notwithstanding that the ultimate raison d’être of any medicine is to benefit patients who have a unique knowledge about their disease and its current therapeutic environment, traditionally patient views and preferences were rarely explicitly sought.

Recent developments, however, have suggested a culture shift is taking place as health care systems and decision-makers have come to realise that the understanding of a disease and of the added value of new medicines could not be fully achieved without engaging with patients (and their caregivers). Roadmaps, processes and tools to enhance patient involvement in drug development [3, 4], medical decision-making [5–9], health technology assessment [10], or drug licensing decisions are now being debated. It still remains unclear, nonetheless, how the patient voice may formally be brought into decision-making beyond anecdotal approaches (such as including one patient on a committee or collecting a handful of testimonials) and how rigorous patient evidence may be generated to be readily accepted by decision-makers. Acknowledging ‘*the added value of patients in benefit-risk considerations – in that they enrich regulatory decisions by complementing them with the views of those directly affected by regulatory decisions’* [11]*,* the European Medicines Agency (EMA) and the U.S. Food and Drug Administration (FDA) have launched a number of initiatives to start capturing patient views, values and preferences to inform benefit-risk assessment [12–16]. Their underlying objective has been to better align acceptance of risks and uncertainties by regulators with that of patients and with the interests of public health more broadly. As the EMA stated, ‘*an excessive focus on avoiding risks and uncertainties concerning new medicines might be against the interests of patients, delaying or reducing access to potentially life-saving treatments*’ [17].

Taking account of patients’ interests is of particular significance when it comes to rare diseases which are serious, debilitating conditions often characterised by poor prognosis and limited treatment options. In this context, rare disease patients and their representatives have urged regulators to be more permissive and to allow for drugs with greater risk or side effects than traditionally accepted [2, 18–20]. For example, in its draft guidance to the FDA the patient advocacy group Parent Project Muscular Dystrophy stated that ‘*in the absence of any approved treatment for Duchenne at all […], the community [of Duchenne patients and families] has expressed a willingness to accept a certain degree of uncertainty regarding both benefit and risk. Some in the community may be willing to take even greater risk – on account of accelerated rates of progression, or their proximity to loss of a vital function or death*’ [19].

Adopting the perspective of the rare disease community (while acknowledging its wide diversity across 7,000 rare diseases), the main purpose of the present study was to contribute to the on-going discussion about what patients affected by a rare disease and their caregivers may consider ‘of value’ when considering new medicines and to quantify their benefit-risk preferences. Under the assumption that values and preferences are context-dependent, our secondary research objectives were to assess to what extent responder-reported assessments of unmet need, disease severity and impairment influence the value attached to key features (or attributes) of future hypothetical treatments. This research used a discrete-choice experiment to assess patient and caregiver preferences across a range of rare diseases.

## Methods

### Discrete-choice experiments

Discrete-choice experiments (DCE) have been used increasingly in recent years to explore and quantify the relative importance of the benefits and risks of different treatments to patients and other stakeholders [21–30]. Well anchored in both psychology and economic theory DCEs offer several advantages over simple rating or ranking exercises, notably because they are shown to give more reliable results [31, 32]. The main postulate of DCEs is that treatments are composed of a set of features, or ‘attributes’ and that the relative value of a particular treatment to an individual is a function of these attributes [33–35]. DCEs can not only consider those treatment attributes specifically related to health outcomes such as efficacy and safety but also those that are process related such as treatment duration, care location or impact on caregivers. In a DCE, respondents are presented with a series of trade-offs in which they are forced to state their preferences by choosing a preferred alternative from a set of hypothetical treatment profiles. These treatment profiles vary by ‘levels’ of treatment attributes that may define the magnitude, severity, likelihood, or timing of each. Statistical analysis of the choices made reveals the implicit relative importance of the attributes of the treatment. The result is an estimate of the perceived value of a treatment as a weighted sum of the treatment attributes.

### Study sample

The design and implementation of this study relied on a partnership with a sample of UK-based patient organisations and support groups, whose activity is targeted at patients and families affected by rare diseases. Eligible study partners were identified and recruited through Orphanet UK’s network of patient organisations. Study participants were subsequently recruited from the study partners’ members list. Eligible study participants were at least 18 years of age, living in the United Kingdom, and were either individuals affected by a rare disease or individuals acting as informal caregivers to individuals affected by a rare disease. Respondents who did not meet the inclusion criteria were screened out. Respondents who did not complete the survey or who always picked the first or last alternative in a choice set were excluded from the sample. Full details on study recruitment flow are available in Additional file 1: Appendix A.

Patients and caregivers were informed that neither benefits nor risks were derived by participating in this study and provided informed consent. The information sheet, consent form, and study protocol were reviewed and approved by King’s College London’s Biomedical Sciences, Dentistry, Medicine and Natural and Mathematical Sciences Research Ethics Subcommittee. For each completed and valid survey, a donation of £2 was made to the participating patient organisations.

### Survey instrument & experimental design

DCEs rely on survey instruments to elicit preferences. A web-based survey, tailored to the two study target audiences (i.e. patients, informal caregivers) was developed. While patients were invited to respond to this survey in light of their own experience of the disease and of their own values and preferences; caregivers, in contrast, were requested to adopt a ‘proxy-patient perspective’ [36] whereby they, as proxies, were asked to respond as they think the patient they usually provide informal care to would respond. The survey had a similar structure for both study target audiences and consisted of three successive modules.

In the first module, respondents had to describe their experience with the disease (e.g. time since diagnosis, current disease management, satisfaction with current care etc.), and were subsequently invited to assess overall disease severity. For this latter purpose we chose to implement three alternative measurements: two Likert scales (range 0–10) capturing self- and proxy-assessed threat to life caused by the rare condition and disease-induced impairment; and the 12-item World Health Organization Disability Assessment Schedule 2.0 (WHODAS 2.0). WHODAS 2.0 is a generic health and disability assessment tool designed according to the International Classification of Functioning, Disability, and Health (ICF) framework [37, 38] [see Additional file 2: Appendix B and Additional file 9: Appendix I].

The second survey module consisted of the DCE that comprised a series of treatment-choice questions defined by seven attributes: (1) ‘*chance that the medicine will work*’, (2) ‘*expected health improvement*’, (3) ‘*risk of experiencing moderate side effects affecting quality of life*’, (4) ‘*additional risk of getting serious side effects leading to life-threatening consequences*’, (5) ‘*treatment duration*’, (6) ‘*burden of treatment*’, and (7) ‘*ability to conduct usual activities while on treatment*’. Three levels were assigned to each attribute [see Additional file 3: Appendix C for details]. The choice of treatment attributes, definitions and levels were informed by peer-reviewed scientific literature, patient advocacy literature, and consultation with patients affected by a rare disease. Our design choices were presented to representatives of the participating patient organisations for validation. Clinical experts also reviewed the choice of attribute levels to ensure that they spanned the clinically relevant range of outcomes that has been seen or may likely be seen in clinical practice.

Attributes and levels were combined to create hypothetical medication profiles. A D-efficient design was generated with 40 choice sets in four orthogonal blocks, each block containing 10 pairs of hypothetical medication profiles. Participants were then randomly assigned to one of the four survey versions. Within each version, the order of treatment choice questions was randomised across participants. More information on the experimental design is included in Additional file 4: Appendix D. During the DCE task, survey participants were asked to point out which hypothetical medication profile in each pair they would choose if the two treatments shown were the only options available. Before completing it, participants were presented with a detailed description of all the attributes and levels by using pictures specifically designed for this purpose in a user-friendly format. Respondents were told to imagine that diagnosis had just happened, that two alternative treatments existed, and that they were in a position to influence the choice of treatment. However, we confirmed that these treatments do not currently exist and assured respondents that this ‘was not a test’ and that there were ‘no right or wrong answers’.

The third and last survey module included socio-demographic questions and an open-text comment field question where participants were invited to bring their own definition of the ‘value of a medicine’.

A pilot study (June 17th–July 5th 2014; *n*: 68) across patients, patient representatives and other healthy volunteers evaluated survey length, logic, skip patterns, trade-off complexity, and wording. Survey length, flow, and language were subsequently amended to address the feedback received. An optional paper version of the survey was made available to participating patient organisations where it was felt that the sole use of a web-based interface may introduce a selection bias as a result of uneven access to/use of Internet (e.g. lower income populations, elderly people).

### Data analysis

Descriptive statistics were calculated for socio-demographic, disease, and care management history variables for all participants. The WHODAS total score was calculated as the sum of the 12-item scores according to the simple scoring instructions [38], with higher scores reflecting greater disability. Summary scores on the reported level of disease-induced impairment and threat to life were analysed. Scores were generated for the variables related to satisfaction with current care, current disease management and impact of care on daily life across the sub-group of participants who had reported on-going treatment for the rare condition. An exploratory unmet need score was derived by summing up the respective ratings of these three variables.

Based on the trade-offs generated through the DCE, a mixed logit model (or random parameter logit model) was estimated with Hierarchical Bayes methodology using the R-package bayesm. As highly flexible models, mixed logit models are considered to be the state-of-the-art discrete choice models [31, 32, 39–44]. They allow to model the variation in preferences among the respondents and yield not only estimates for the mean preference and preference heterogeneity, but also individual estimates representing the value that each respondent attaches to the different attribute levels. They are therefore best suited to investigate the relationship between the individual preferences and various covariates. The importance of each attribute was computed for each respondent as the difference between the individual part-worth estimate of the first (best) attribute level minus the individual part-worth estimate for the last (worst) attribute level (truncated below at zero if necessary) and then rescaled by the sum to obtain the individual relative importance value for each attribute. Tests for significance were performed on the individual part-worths and relative importance for each attribute and the robustness of these results was checked thoroughly as explained in Additional file 5: Appendix E.

To evaluate whether the relative importance values are influenced by the context of the disease – as captured by respondents’ reported assessments on disease-induced impairment, disability, threat to life, and overall unmet need – a series of regression analyses where run where the individual relative importance values for each attribute were related to these measures in the total sample and different subsamples to check the validity of the results.

Lastly, patients’ open-text comments on the ‘value of a medicine’ were reviewed: statements were categorised in terms of their specific focus and were then grouped into broad themes.

## Results

### Study sample

The survey was administered between August and November 2014 across 16 UK-based patient organisations – collectively representing over 80 rare/genetic conditions – which had agreed to partner this research project. At closure, the survey had yielded 1,160 responses and 893 participants qualified for the survey: 17.2 % had dropped out from the survey before answering any trade-off question and 5.8 % did not complete the trade-off module or survey. Twenty further observations were excluded from the dataset after internal validity checks showed that those respondents had always selected either the first or last alternative in each choice set [see Additional file 1: Appendix A and Additional file 5: Appendix E for further details]. The profiles of respondents who were included and excluded in the analysis were similar in terms of disease and socio-demographic characteristics.

After these exclusions, 873 respondents were available for the final analysis: 721 patients (82.6 % of total sample) and 152 informal caregivers (17.4 %), across a total of 52 rare diseases. Additional file 6: Appendix F presents summary statistics for the 873 respondents included in the final analysis. Just over two-thirds of participants were female (67.5 %), Caucasian (87.9 %) and half the participants were aged between 35 and 54 years. Overall, the three most represented groups of diseases were: ‘rare systemic or rheumatological diseases’ (49.6 %), ‘rare neurologic diseases’ (19.0 %), and ‘inborn errors of metabolism’ (17.9 %). Circa 70 % of respondents concentrated across 10 rare diseases, namely: sarcoidosis (*n*:418), Fabry disease (*n*:36), Duchenne muscular dystrophy (*n*:26), hypopituitarism (*n*:26), transverse myelitis (*n*:26), amyotrophic lateral sclerosis (*n*:24), Pompe disease (*n*:19), autosomal dominant polycystic kidney disease (*n*:18), McArdle’s disease (*n*:16), and mucopolysaccharidosis I (*n*:16).

Over a third of the surveyed patients (36.5 %) had been diagnosed over ten years ago and 70.7 % of patients (*n*:510) reported being on treatment for their condition. While 27.3 % of patients were employed full-time, a similar proportion of patients declared being unable to work due to disease-induced disability.

Caregivers were predominantly women (83.6 %) and mostly cared for patients affected by inborn errors of metabolism or rare neurologic diseases. 82.9 % (*n*:126) of the patients they provide informal care to were reportedly under treatment. Within our sample, one out of five caregivers had to stop working to provide informal care. Whilst most caregivers were providing care as a parent (63.8 %), they were almost equally split between caregivers to a child (48.7 %) and caregivers to an adult (51.3 %). Nearly 40 % of them had been providing informal care for over ten years, and spending over 60 h per week to perform that commitment.

The WHODAS 2.0 distribution of mean scores across our study sample suggests clinically significant disability. While the mean for the patient respondents was 16.6 (SD: 11.7, range 0–48) and that for the caregiver proxies was 26.2 (SD: 13.4), it is noteworthy that only 5 % of responding patients scored 0 – that is, reported no difficulty in any activity. In contrast, 31 % (*n*:223) scored above 24 and 6 % (*n*:46) above 36. Although there is no agreed upon cut-off point for identifying individuals with significant disability [45], scores ranging 24–35 and those above 36 are likely to represent moderate-to-severe and severe-to-extreme disability respectively. The fact that caregivers scored higher than patients seems logical since most caregivers from our study sample concentrate across some of the most debilitating conditions, such as Duchenne muscular dystrophy, amyotrophic lateral sclerosis, or Sanfilippo disease [see Additional file 6: Appendix F]. The other measurement tool used in our survey related to impairment assessment on a scale ranging 0–10 yielded a similar picture. Of note, correlation between these two scales was found to be 78.2 %.

When invited to assess their life expectancy compared to that of other individuals of the same age and gender but unaffected by the rare condition, 19 % of patients (*n*:134) reported they would expect to live as long as them; 11 % of patients (*n*:82), however, believed they would have a ‘much shorter life’. The opposite was observed from assessments from caregivers, as 51 % of them were of the opinion that the patients they provide informal care to would die earlier. The box-and-whisker plots available in Additional file 6: Appendix F provide further details on the distribution of the various assessment scores used in this study.

As shown in Table 4 in Additional file 6: Appendix F, ca. 45 % of respondents declared being satisfied with current care, with other 25 % being dissatisfied. Owing to the paucity of therapeutic alternatives to manage rare diseases, only 10.6 % of participants reported an improvement in health condition brought by current care or a cure from the rare disease. Current disease management was reported to result in: no improvement to health condition (10.8 % of respondents); moderate survival extension (8.3 %); improvements in quality of life (18.6 %); symptom management (30 %); disease stabilisation (21.7 %).

### Relative importance scores, preference weights & risk-benefit trade-offs

**Fig. 1:**
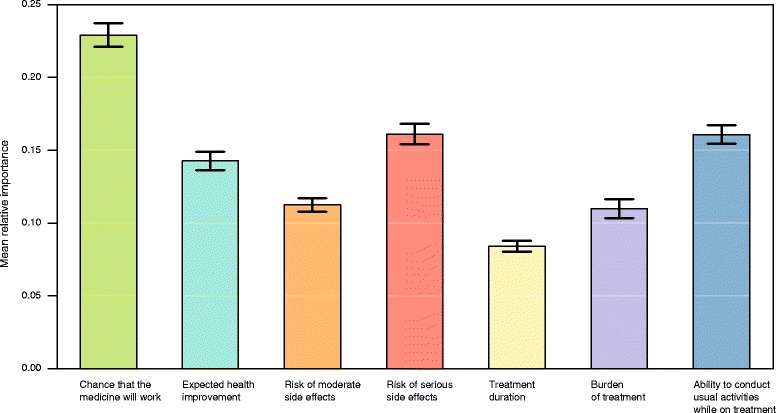
Attribute mean relative importance scores (*n*: 873). Legend: The black vertical bars denote the two-standard-error intervals around mean relative importance estimates

Figure 1 presents for each attribute the mean relative importance value and corresponding two-standard-error interval. The most important attributes of a new medicine for patients and caregivers across our study sample were: ‘*the chance that the medicine will work*’, followed by ‘*the risk of serious side effects*’ and ‘*the ability to conduct usual activities while on treatment*’ (which were both valued equally high), and the ‘*expected health improvement*’ that the medicine may bring. The least important attributes proved to be ‘*treatment duration*’, followed by ‘*burden of treatment*’ – corresponding to two out of the three attributes relating to process of care.

**Fig. 2:**
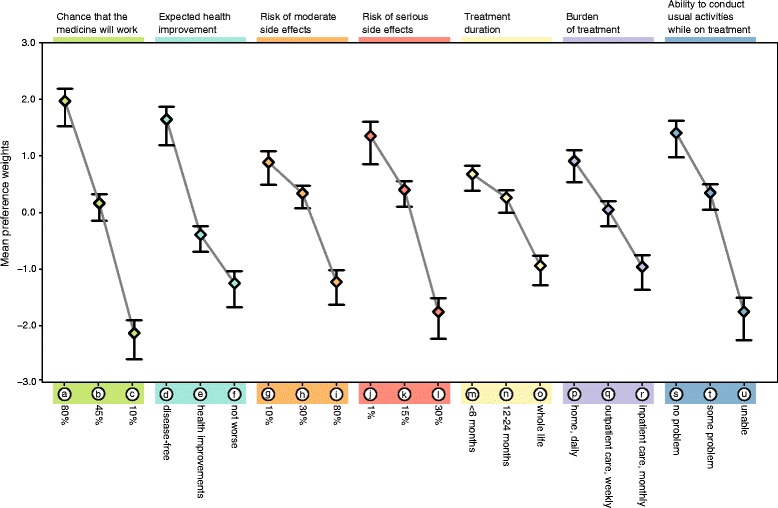
Mean preference weight per attribute level (*n*: 873). Legend: Treatment attribute labels are shown at the top of the graph; treatment attribute level descriptions at the bottom. The black vertical bars around mean preference weights denote the two-standard-error intervals

Figure 2 shows the estimated mean preference weight (or part-worth) for each attribute level with the respective two-standard-error interval. The intervals did not overlap, inferring that mean estimates can be considered as statistically different between all the adjacent levels. In general, preferences for the levels under each treatment attribute were consistent with the a priori expectation that levels with better outcomes, lower toxicity, or lower care burden have higher preference weights (i.e. they are more preferred) than levels corresponding to worse outcomes, higher toxicity or greater care burden. Please note that the absolute scale of the part-worths is arbitrary; only relative differences across attributes and attribute levels are meaningful.

This graph conveys several messages.

First, the difference between adjacent importance weights indicates the relative importance of moving from one level of an attribute to an adjacent level of that attribute – the greater the distance, the greater the importance attached to moving from one level to the next. For instance, data suggested that, on average, respondents attached a greater importance to moving from a high risk to a moderate risk of serious side effects [see change from l to k] than to reducing that risk from a moderate to a low level [from k to j]. In contrast, respondents assigned the same value to the opportunity to shift from a monthly inpatient care setting to a weekly outpatient care setting [r to q], as to moving from weekly outpatient care to daily care delivery at home [q to p] and vice-versa.

Second, and most importantly, the difference between adjacent importance weights of one attribute can be compared with the difference between adjacent importance weights of another attribute – thus informing us on possible trade-offs. On average, respondents were willing to accept a 70 % increased risk of facing moderate side effects [g to i] in exchange for a 35 % increased chance to respond favourably to a new medicine [c to b]. Also, to get the opportunity to shift from a state of stabilised disease to actual gains in overall functioning and symptom alleviation [f to e], respondents showed a willingness to accept a 20 % increase in the risk of moderate side effects [g to h] or a 14 % increase in the risk of serious side effects [j to k]. Similarly, within the hypothesis that they would be able to move away from a state of improved functioning to a cure of the disease [e to d], respondents would be ready to give up all of their ability to conduct daily activities while on treatment [t to u], or to face an increase in the risk of serious side effects from 15 to 30 % [k to l].

In summary, when considering the opportunity of a new medicine targeting a rare condition, patients with rare diseases and their caregivers stressed that they attributed most importance to drug response, risk of serious side effects, and the ability to conduct usual activities while on treatment. However, our study suggested that they were prepared to trade a significant amount of risk associated with a new medicine for a higher chance of drug response, or greater health improvement potential.

### Effect of respondent-reported disease burden assessment on attribute relative importance scores

A secondary objective of our study was to explore whether ‘disease context’, as measured by respondent-reported assessments on disease-induced impairment, disability, threat to life, satisfaction with current care, current disease management and unmet need had an influence on the relative importance of these attributes.

**Fig. 3:**
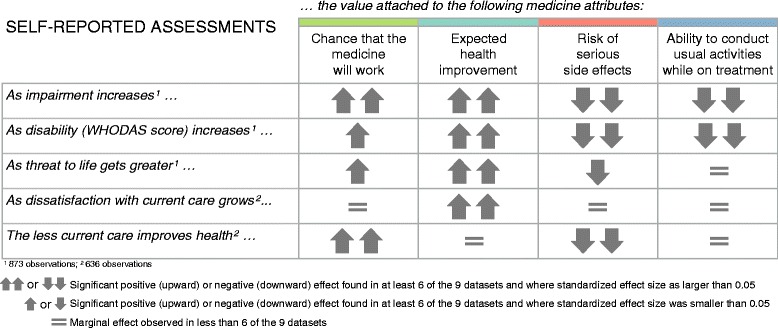
Influence of disease context on attribute importance. Legend: This figure summarises the regression analyses investigating the effect of disease context on the relative importance of the four attributes that were found earlier as the most important to respondents. The magnitude and direction of the estimated standardized effects are visualized either by an equals sign or by one or two arrows, representing respectively the absence of effect, a small effect, or a large effect

Figure 3 presents a summary of the regression analyses investigating the effect of disease context on the relative importance of the four attributes that were found earlier as the most important to respondents. The magnitude and direction of the estimated standardized effects are visualized either by an equals sign or by one or two arrows, representing respectively the absence of effect, a small effect, or a large effect. Additional file 5: Appendix E provides more technical details on the statistical methods followed and Additional file 7: Appendix G reports the standardized regression coefficients and related statistics.

Within our study sample across 52 rare diseases, the performed regression analyses described clear relationship patterns between context of disease and attribute relative importance. Data showed that as respondents reported increasing impairment or disability, the value attached to drug response and to health improvement potential increased; in contrast, both the risk of serious side effects and the ability to conduct usual activities became less important to patients or their caregivers. The same findings were observed as the rare disease becomes more life-threatening (with the exception of the ability to conduct daily activities where no relationship was found). Likewise, our data showed that expectations for health improvement brought by a new medicine increase as dissatisfaction with current treatment increases. Lastly, the analysis concluded that the less current disease management improves health condition, the more one is ready to face the risk of serious side effects associated with a new medicine (i.e. the projected value of this attribute decreases); and the more drug response potential becomes important.

An alternative analysis of the regression output – that looked at how often statistical significance and observed effect were found, instead of the observed size of the standardized effect as reported above – yielded similar conclusions. The outcome of this sensitivity analysis can be found in Additional file 7: Appendix G. Of note, the exploratory composite variable related to unmet need proved insensitive to change (whilst its individual components did) and is therefore not shown in Fig. 3. Details on its statistical performance are available in Additional file 5: Appendix E and Additional file 7: Appendix G.

Lastly, we also explored the presence of an effect of disease context on the other three attributes included in our study, namely ‘treatment duration’, ‘burden of treatment’ and ‘risk of moderate side effects’ – that were found the least important attributes for patients and their caregivers. Hardly any effect was found from our review, except to some extent on the attribute related to burden of treatment.

In conclusion, our analysis quantified the influence of disease context on rare disease patients’ and caregivers’ attitudes about benefit-risk. It provides evidence to support a widely shared assumption and a more detailed, robust, understanding of the benefit-risk trade-offs that respondents are ready to make, across different patient populations and respondent types.

### Patients’ open-text comments on ‘the value of a medicine’

Circa a third of survey participants took the opportunity to comment at the end of the survey on their personal experience of living with a rare disease, on their own definition of value and on the survey itself. (Table 1) displays a few excerpts from the qualitative feedback collected from patients specifically. More quotations are available in Additional file 8: Appendix H. Overall, five main themes of comments emerged: hope, attitude to risk, healthcare professionals, steroids use, and introspection. All of these qualitative insights – stemming from patients who have learnt to live with a poorly understood, serious and progressing condition with limited treatment options – bring both real-life anchor and colour to the quantitative data that our study generated.

**Table 1:** Patients’ open-text comments on the ‘value of a medicine’ (excerpts)

On hope
* ‘To have no hope is to have no life or anything to live for – any chance […] of not just a cure but hope that life will be longer or will not deteriorate makes a difference beyond anything.’* * ‘Any new medicine that can give some hope to those suffering with such diseases is invaluable.’* * ‘I would prefer research into new medicines [addressing my main symptoms], rather than research into a ‘cure’ for my condition.’* * ‘We live in hope.’* * ‘Any port in the storm.’*
On risk
* ‘With motor neurone disease any improvement outweighs any side effects. Better to die than live with this cruel disease.’* * ‘We have nothing to lose but everything to gain.’* * ‘As I am symptom-free at present, I am against health risks. However, once my condition deteriorates, my attitude to health risks will change.’* * ‘[Minor] side effects should not be underestimated or played down. [They] can be extremely wearing and challenging when they occur every day.’* * ‘I have discovered I am better off with no medicine, the side effects made me feel worse.’*
On interactions with healthcare professionals
* ‘Regular contact with health care professionals […] is invaluable.’* * ‘Doctors need to give us the information so we can decide for ourselves.’* * ‘Health professionals take a very ‘paternalistic’ stance and give minimum information – “they know best”.’* * ‘Each new medicine should have the side effects explained in percentages like this survey.’* * ‘Anything that engages the sufferer in discussions determining how to handle treatment/medication can only be beneficial to the patient.’*
On the impact of long-term use of steroids
* ‘[The medication] I object to most are steroids because of the physical changes they have made to me. This side effect may not be as relevant/important to other patients. This is an area the medical profession need to be more sympathetic towards.’* * ‘Doctors prescribe [steroids] and do not explain the side effects and long term effects and risks without discussing it with you or giving you the options. They got me into remission after two years but I am still suffering with other diseases caused by [steroids]. I would have opted for other options if I was given a choice.’*
Patients’ introspection
* ‘Really interesting survey, I learnt a lot about my attitude to risk.’* * ‘I found the survey quite thought provoking and made me think about my current treatment plan and other options facing me.’* * ‘Answering the questions about value made me realise I regard the chance of a beneficial effect as outweighing any* *possible risk or discomfort.’*

Note: More patient quotations can be found in Additional file 8: Appendix H

Confronted with the ever approaching outcome of disease progression, impairment or death, patients made a compelling statement of ‘hope’: hope for new medicines, hope for a halt to disease progression, hope for symptom alleviation or partial recovery. In the context of genetic diseases where a cure may reportedly be impossible (at least within their life span), very few patients stated being actually hopeful for a cure or full recovery. This pragmatic stance balancing hope with self-managed expectations may explain why survey respondents during the quantitative trade-off scenarios assigned a greater value to drug response potential than to the actual magnitude of health improvement brought by a new medicine.

A corollary to this ‘pragmatic hope’ is a willingness to take risks. When weighing uncertain benefits brought by a new medicine with unknown risks or outcomes, patients showed an articulate and informed attitude to risk that they modulate according to their personal circumstances of disease seriousness, disease stage, or current disease management. Whilst some patients declared having ‘nothing to lose but everything to gain’ and thus being ready to face serious side effects, unknown outcomes or even death in the hope that a treatment might offer them some benefit, others stated being more reserved. This correlation between risk readiness and disease context expressed across these patient statements echoes our quantitative findings shown in Figs. 2 and 3.

Patients also suggested an eagerness to be actively involved in their own care and to become joint decision-makers in treatment choices. They, however, report an ambivalent relationship with their treating doctors and other healthcare professionals. While ‘regular contact with [doctors] is invaluable’, the latter may prove paternalists and may not always listen to the patient’s feelings or treatment preferences. ‘Doctors need to give us the information so we can decide for ourselves’, some patients reported. The case of high-dose steroids use was mentioned by a number of patients, where they felt they had not been duly informed of their induced comorbidities at time of treatment decision.

## Discussion

Rare diseases are poorly understood, serious, debilitating conditions often characterised by poor prognosis and limited treatment options. Because patients and caregivers constantly navigate the health care system in search of disease management, they gradually gain an intimate knowledge of the rare condition and are thus in a position to identify when a treatment outcome becomes ‘meaningful’ to them and to subsequently articulate trade-offs.

Within the boundaries of our study, rare disease patients and their caregivers chose to adopt a realist view and attributed the highest importance to drug response potential, highlighting the importance of developing new targeted therapies, diagnostic tests and expanding the use of biomarkers [46–49] to better predict health benefit. We may also interpret this finding as a result of ‘pragmatic hope’, as discussed earlier. Next, the other two dimensions of highest value were the risk of serious side effects and the ability to conduct usual activities while on treatment. We consider the latter finding regarding patients’ activities compelling since it is usually overlooked by researchers and clinicians who tend to focus on the pathophysiological mechanisms and consequences of a disease rather than on how the patient feels and lives on a daily basis. In contrast, attributes related to treatment modalities (i.e. how long, where and how to take the medicine) were deemed the least important, although the latter tend to be the focus of many drug development programmes.

Additionally, our study data confirmed that patients and their caregivers were willing to accept greater risk or side effects associated with a new medicine, for instance, in the hope for some extra chance in drug response or greater health improvement potential. And, as demonstrated by the outcome from the regression analyses performed, attitudes about benefit-risk may change over time with disease progression or context of care. Our findings are consistent with the analysis by social scientists who defined risk as a ‘social construction’ rather than an objective and measurable function of the probability and magnitude of an event [50, 51]. As Douglas pointed out, ‘*risk is not only the probability of an event but also the probable magnitude of its outcome, and everything depends on the value that is set on the outcome. The evaluation is a political, aesthetic and moral matter*’ [52]. We may wish to add the word ‘*contextual’* to that definition to bring in the dimensions of unmet need and disease progression.

Overall, our research findings – which are reminiscent of the work by Kesselheim [53] or Peay [54] – highlight the need to systematically include patients in the process of identifying meaningful treatment outcomes that resonate with their experience, preferences, expectations and values. As stated by Dr. Janet Woodcock from the U.S. FDA ‘*it is clear you have to start with an understanding of the impact of the disease on the people who have it, and what they value most in terms of alleviation before you set up a measurement and go forward with truly patient-focused drug development*’ [55].

In that context, we believe that patients and caregivers should be increasingly involved as active research partners in the development of clinical outcomes assessments – including patient-reported outcomes (PRO) measures – that directly evaluate how the patient feels, functions or survives [56–58]. If a treatment effect is not meaningful to the patient, it is not a benefit to the patient. Additionally, we believe that patient preference data informing the development of those assessments should be treated with the same level of scientific rigour as currently with clinical data. Despite their different focus, if guided by a clear hypothesis-led strategy, both patient preference and PRO data offer complementary evidence to substantiate label claims of ‘significant benefit’ or ‘major contribution to patient care’. From that perspective, researchers of novel therapeutics for rare diseases should be encouraged to invest in the use of such methods and specific regulatory guidance should be issued to acknowledge their importance and to state where these methods may fit into drug development programmes and regulatory decision-making.

Patient-centred outcomes are key complements to traditional clinical data that can purposefully help detect meaningful and interpretable changes in health status and treatment benefit. Efforts from the research community to promote the development and use of patient-centred outcome measures in rare diseases, such as the current work by the International Rare Disease Research Consortium (IRDiRC) are, in that context, promising [59].

### Limitations

This study includes a number of limitations; the four main ones are discussed here. First, as a result of its ambition to review the preferences and values across over 50 rare conditions, the trade-offs module of our survey applied non-disease-specific, generic attributes. This limitation in the design of our study, however, was reviewed and validated by our partners from the patient organisations, and the survey was deemed appropriate for use across different disease profiles. Second, our analysis relied on observations across a subset of rare diseases – 52 of them – and an uneven number of patients by disease, in part reflecting differences in prevalence. Additionally, the diseases included in our study are very different from each other and variations within each disease are also observed. The reality of that limitation was acknowledged at time of study design and justified the use of the three alternative self- / proxy-reported assessment measurements to address it. In addition to the individual estimates yielded from our DCE model, individual differences in disease severity across the variety of diseases were thus duly taken into account in our regression analyses. Third, our study sample is limited to patients and caregivers living in the United Kingdom. Since patient autonomy has become the dominant principle shaping physician-patient relationships but remains culture-dependent, our study results may not apply as a proxy for patient preferences in another national healthcare setting. Fourth, our analysis relied on the pooled data of patients’ and caregivers’ preferences: comparative results between respondent types validated that choice.

## Conclusion

To the best of the authors’ knowledge, this study – made possible thanks to the sustained commitment from sixteen UK-based patient organisations – is the first study performing a quantitative discrete choice experiment amongst patients and their caregivers across 52 rare conditions.

Conceptually anchored to the overarching goal of incorporating the patients’ perspectives into drug development and approval processes, our study aimed to better understand and quantify the preferences and values of rare disease patients and families and to explore to what extent the specific context of disease – such as disease-induced disability or the perceived medical added-value of currently available treatments – may alter these preferences and values. This study offers an alternative approach to generate robust evidence about patients’ preferences and values from a wide range of patients that can ultimately input to complete drug development and approval decision-making processes.

## Additional files

Additional file 1: Study Recruitment Flow & Study Sample. (DOCX 56 kb)

Additional file 2: Self-/ Proxy- Reported Instruments used in the survey. (DOCX 67 kb)

Additional file 3: Attributes and Levels used in the DCE. (DOCX 23 kb)

Additional file 4: Experimental Design. (DOCX 24 kb)

Additional file 5: Tests of Internal Validity & Statistical Analysis. (DOCX 23 kb)

Additional file 6: Study Sample. (DOCX 133 kb)

Additional file 7: Mixed logit estimates & regression output. (DOCX 186 kb)

Additional file 8: Patients’ quotations on ‘the value of a medicine’. (DOCX 25 kb)

Additional file 9: References used in supplementary material. (DOCX 21 kb)
