# Supplementary material for: Quantifying benefit-risk preferences for new medicines in rare disease patients and caregivers
Source: Orphanet J Rare Dis. 2016 May 26;11:70. doi: 10.1186/s13023-016-0444-9 (PMC4881055; doi:10.1186/s13023-016-0444-9)
Supplement: Supplementary file 1 — Study Recruitment Flow & Study Sample. (DOCX 56 kb) [file 13023_2016_444_MOESM1_ESM.docx]

Appendix A – Study Recruitment Flow & Study Sample

RECRUITMENT OF PARTNER PATIENT ORGANISATIONS

*[Jan-Feb 2014]*

Call for interest to patient organisations (POs) across Orphanet UK to participate in planned study
(n: 315)

Declined participation

*(n: 7)*

POs answered call of interest

*(n: 69, 22% of total)*

Excluded from study participation, with reasons

*(n: 30)*

*Membership size <100, n = 13*

*Other exclusion criteria, n = 17*

Review of POs for eligibility

*(n: 62)*

*[April 2014]*

Formal invitation to POs to participate, with study information package

(n: 32)

*[June 2014]*

POs confirmed study partnership

*(n: 16)*

RECRUITMENT OF STUDY PARTICIPANTS

Excluded from dataset,

with reasons *(n: 267)*

*(Patients, n: 163; Caregivers, n: 46; Unknown, n: 58)*

*Early survey drop-out, n: 58*

*Drop out at time of conjoint module, n: 142*

*Did not complete the conjoint module, n: 54*

*Did not complete survey, n: 13*

*[Aug. 4th – Nov. 15th 2014]*

Study recruitment (i.e. patients & caregivers)

1,160 study participants

*Patients, n: 902*

*Caregivers, n: 200*

*Unknown, n: 58*

**893 eligible responders**

*Patients, n: 739*

*Caregivers, n: 154*

INTERNAL VALIDITY

Excluded from dataset,

*(n: 20)*

*(Patients, n: 18;*

*Caregivers, n: 2)*

**Final sample, n: 873**

*Patients, n: 721*

*Caregivers, n: 152*

Internal validity tests between included (n: 873) and excluded (n:181) observations

Study recruitment was organised in successive phases.

First, study partners were sought across Orphanet UK’s network of patient organisations. Upon response to a preliminary call for interest sent to 315 patient organisations, possible candidates for study partnership were screened for eligibility (n=62). Eligible candidates to study partnership had >100 members; represented ‘life-threatening, seriously debilitating or serious and chronic conditions’; and did not: (i) cover rare cancers, (ii) exclusively represent neonatal conditions, (iii) represent conditions with a prevalence level > 5 per 10,000, or (iv) represent rare conditions that may be a comorbidity resulting from a more common condition. The 32 pre-selected patient organisations were subsequently provided with a full information package on the planned study and were invited to state their interest in becoming study partner by the end of June 2014.

Sixteen patient organisations, collectively representing over 80 rare/genetic conditions, agreed to partner with the researchers and to facilitate study recruitment by promoting the planned study through their usual communication channels (i.e. emails, newsletters, websites, social media, or paper communication formats) across their members (i.e. patients and caregivers).

The survey was rolled-out between August 4^th^ and November 15^th^ 2014. Eligible survey participants were at least 18 years of age, living in the United Kingdom, and were either individuals affected by a rare disease, or individuals acting as informal caregivers to someone affected by a rare condition. Respondents who did not meet the inclusion criteria were screened out. Respondents who did not complete the survey or who always picked the first or last same alternative were excluded from the sample.

*Note*: In addition to their role in study recruitment, participating patient organisations were consulted on the design of our survey. In particular, senior representatives from the sixteen patient organisations were consulted on the choice of treatment attributes, definitions and levels and also on survey length, complexity, and language. Seven out of them made comments, which were then reflected into our study design. Due to its technical nature, patient representatives were not involved in statistical data analysis. Overall study results will be communicated to them in lay terms in a separate document to allow them inform their members.
