# Supplementary material for: Quantifying benefit-risk preferences for new medicines in rare disease patients and caregivers
Source: Orphanet J Rare Dis. 2016 May 26;11:70. doi: 10.1186/s13023-016-0444-9 (PMC4881055; doi:10.1186/s13023-016-0444-9)
Supplement: Supplementary file 2 — Self-/ Proxy- Reported Instruments used in the survey. (DOCX 67 kb) [file 13023_2016_444_MOESM2_ESM.docx]

Appendix B – Self-/ Proxy- Reported Instruments used in the survey

**Likert scale on threat to life caused by the rare condition, score range 0 – 10**

(the example below was used in the survey targeting patients)

*“Compared with someone of the same age and gender as you but WITHOUT your condition, how much is your life in danger because of your condition?”*


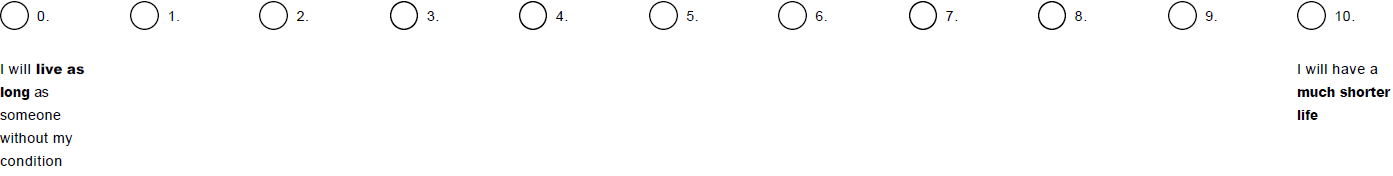


**Likert scale on impairment level, score range 0 – 10**

(the example below was used in the survey targeting patients)

*“What is the level of impairment induced by your condition?”*


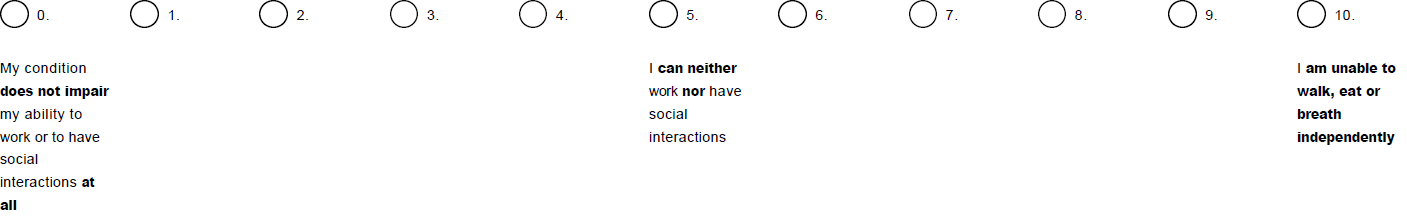


**World Health Organization Disability Assessment Schedule 2.0 (WHODAS 2.0), score range 0 – 48**

WHODAS 2.0 is a generic health and disability assessment tool designed according to the International Classification of Functioning, Disability, and Health (ICF) framework. It describes effects of disease on six domains: cognition, mobility, self-care, getting along, life activities, and participation in society. In each questionnaire item, individuals have to assess the magnitude of disability over the previous 30 days using a five-point scale (none = 0, mild = 1, moderate = 2, severe = 3, extreme/cannot do = 4). In the 12 item version of the WHODAS 2.0, total score is computed as the sum of the 12 items (range 0 – 48), with higher scores reflecting greater disability. WHODAS 2.0 has been tested for its psychometric properties in a number of studies across different population samples and health conditions [1-6]. Administration time is less than 5 min.
