# Supplementary material for: Quantifying benefit-risk preferences for new medicines in rare disease patients and caregivers
Source: Orphanet J Rare Dis. 2016 May 26;11:70. doi: 10.1186/s13023-016-0444-9 (PMC4881055; doi:10.1186/s13023-016-0444-9)
Supplement: Supplementary file 3 — Attributes and Levels used in the DCE. (DOCX 23 kb) [file 13023_2016_444_MOESM3_ESM.docx]

Appendix C – Attributes and Levels used in the DCE

| **Attribute labels** | **Definition displayed in survey** | **Levels** |
| --- | --- | --- |
| **Chance that the medicine will work** | A disease may have many different forms and every individual affected by a disease is different from another individual affected by the same condition. As a result, a medicine that works for one individual may not work for another.  In this survey, we will describe three possible responses to a medicine: [*graphical representation of the three attribute levels*]. | - very good chance (80%) - moderate chance (45%) - small chance (10%) |
| **Expected health improvement**  (abbreviated in survey as: ‘How the medicine will improve health’) | A medicine may have a strong, medium or moderate impact on a disease. For instance:   - a medicine may clear a disease from an individual’s body; or - a medicine may aim to reduce the level of symptoms induced by a disease (e.g. pain); or - a medicine may help patients’ health not to deteriorate as fast as initially predicted (in the absence of treatment) (e.g. a treated patient will not need a transplant anymore; or time to wheelchair is postponed by 5 years).   In this survey, we will describe three possible levels of health improvement: [*graphical representation of the three attribute levels*]. | - the treated patient will live disease-free - the treated patient will see improvements in symptoms & functioning - the treated patient will not get worse |
| **Risk of experiencing moderate side effects, affecting quality of life (e.g. pain)**  (abbreviated in survey as: ‘Risk of moderate side effects’) | Side effects are unwanted symptoms caused by a medicine. Sides effects may be:   - "mild", and temporary (e.g. nausea) - "moderate", and affecting quality of life (e.g. pain); or - "serious", that may have life threatening consequences (e.g. heart failure).   The risk of getting side effects varies from individual to individual. Every patient meeting his/her treating physician is usually informed about the benefits of a treatment and warned against its possible risk of side  effects. In this survey, we will ask you to think about new medicines with different risks of getting "moderate" or "serious" side effects.  [*graphical representation of all attribute levels*]  The following pictures may help you think about this risk. Each person in the boxes below represents one person who takes a medicine. There are 100 people in each box. The people marked by colour will experience side effects. The people shaded grey will not. | - small risk (10%) - high risk (30%) - very high risk (80%) |
| **Additional risk of getting serious side effects, leading to life-threatening consequences (e.g. heart failure)**  (abbreviated in survey as: ‘Risk of serious side effects’) |  | - very small risk (1%) - moderate risk (15%) - high risk (30%) |
| **Treatment duration** | A treatment may take place at home, or during a hospital stay. It may be more or less burdensome, depending on 'how', 'where' and 'how often' treatment is delivered.  A treatment may negatively impact on patients’ (or caregivers’) ability to conduct usual activities, or not.  Overall treatment duration may be brief or lifelong.  In this survey, across various scenarios, we will ask you to think about treatment burden, treatment duration, and ability to conduct usual activities.  [*graphical representation of all attribute levels*] | - Six months or less - Between 12 and 24 months - Your whole life |
| **Burden of treatment**  (abbreviated in survey as: ‘How to take the medicine’) |  | - at home every day - during a same-day hospital visit every week - during a hospital stay of one week per month |
| **Ability to conduct usual activities while on treatment** |  | - no problem with performing usual activities - some problems with performing usual activities - unable to perform usual activities |
