# Supplementary material for: Quantifying benefit-risk preferences for new medicines in rare disease patients and caregivers
Source: Orphanet J Rare Dis. 2016 May 26;11:70. doi: 10.1186/s13023-016-0444-9 (PMC4881055; doi:10.1186/s13023-016-0444-9)
Supplement: Supplementary file 4 — Experimental Design. (DOCX 24 kb) [file 13023_2016_444_MOESM4_ESM.docx]

Appendix D – Experimental Design

The design of a discrete choice experiment, i.e. the choice of the profiles and the combination of the profiles to be shown together to the respondents, is an important aspect of discrete choice experiments as it determines the models that can be estimated and the precision of the parameter estimates. An inefficient design is not only costly (more respondents and/or choice sets are needed for a specific level of accuracy) but can also lead to an incorrect assessment of the mean preference and of the individual preferences of the respondents and therefore also of the heterogeneity in the population [7-11].

For the pilot study we generated 16 choice sets with pairwise partial profiles varying only 5 of the 7 attributes. This design was Bayesian D-optimal for the multinomial logit model with a normal prior distribution with mean value 0.5 for the first level of each attribute and 0 for the second level, all with standard deviation 0.2. As effect-coding was used, this prior reflects that the levels go from best to worst for each attribute. The design was optimized for all main effects and all two-way interactions between the attributes ‘Chance that the medicine will work’, ‘Level of health improvement that the medicine may bring’ and ‘Additional risk of getting serious side effects’ though these interactions were later found not to be significant. Remark that two combinations of attribute levels were not considered feasible and therefore removed from the candidate set: ‘the treated patient will live disease-free’ (first level of second attribute) and ‘a lifetime treatment duration’ (third level of fifth attribute) were considered incompatible with ‘being unable to perform usual activities’ (third level of last attribute).

Based on the results of the pilot study, it was decided to limit the number of choice sets to avoid respondent fatigue and to let all attributes vary in each choice set. The model and prior values from the pilot study were retained. Bayesian D-optimal designs were generated with 40 choice sets in 4 orthogonal blocks, each block containing 10 pairs of hypothetical medication profiles.

Several designs were generated using SAS and Ngene software and the one with the best local D-efficiency for the mixed logit model was selected using the prior distribution as heterogeneity distribution. Respondents were randomly assigned to one of the four blocks and the choice sets of each respondents were shown in a randomized order.
