# Supplementary material for: Quantifying benefit-risk preferences for new medicines in rare disease patients and caregivers
Source: Orphanet J Rare Dis. 2016 May 26;11:70. doi: 10.1186/s13023-016-0444-9 (PMC4881055; doi:10.1186/s13023-016-0444-9)
Supplement: Supplementary file 5 — Tests of Internal Validity & Statistical Analysis. (DOCX 23 kb) [file 13023_2016_444_MOESM5_ESM.docx]

Appendix E – Tests of Internal Validity & Statistical Analysis

We included several tests for internal validity by comparing the results of the statistical analyses on different subsets of the complete dataset. The largest dataset that was considered included 873 respondents (721 Patients and 152 Caregivers) and we report in this paper on the results of that analysis. However, we only report on findings that were confirmed in the different subsets.

As the attribute levels of every attribute were sorted from best to worst and the attribute levels were coded such that the three part-worths corresponding to an attribute sum to zero, the part worth of the first level is expected to be positive, the part worth of the last level is expected to be negative and the part worth of the second level should lie in between the other two estimates. As the measure of the relative importance of an attribute is based on the estimate of the first level from which the estimate of the third level has been subtracted, an extra test of rational choice behavior is to check whether this difference is positive. Based on these four checks we selected sets including only those respondents that satisfied one, two, three or all four of these checks yielding 627, 573, 472 and 298 observations respectively.

A mixed logit model was first estimated based on the choices of all 873 respondents and the different tests were conducted on those estimation results for the 873, 627, 573, 472 and 298 respondents. Separate mixed logit models were estimated on the 4 subsets containing only the choices of the selected respondents and the analyses were repeated on those estimation results. We report here on the results that were found to be significant at the 5% level in most of the versions of a particular test.

We estimated first a multinomial logit model which assumes that all respondents under consideration (whether all respondents or only the respondents of one of the subsets examined) attach the same weight to the different attribute levels. Using interaction terms, we have tested for significant differences of the estimated weights between different subgroups. However, to investigate in a reliable way the relative importance that respondents attach to the different attributes, we need individual weights or part-worths [10], which are obtained by estimating a mixed or random parameter logit model. For each attribute, we computed the mean and corresponding standard error of the estimated individual relative importance values to represent the mean relative importance and its accuracy. We report here only on the tests based on the mixed logit model estimates as they confirm and generalize those that could also be conducted based on the multinomial logit model.

Some descriptive statistics on the posterior distributions of the individual part-worths as estimated on the dataset with the choices of 873 respondents are given in the following table. The estimates for the third level of each attribute could be derived from the estimates of the other levels as the sum of the parameters of each attribute should be zero with the effects coding that was used. For all attributes, the mean estimate for the first level is larger than that of the second level which is larger than the part-worth corresponding to the last and worst level, showing that on average the respondents indeed responded as expected though there were some outliers. The tests were conducted on the complete dataset and on various reduced datasets where some or all of the respondents with irrational choice behavior were removed as explained earlier.

Last, we used regression analysis to assess whether the importance that people attach to the different attributes is context dependent. To this end, we checked for each attribute separately whether a significant part of the variation in the relative importance values could be explained by these context variables. We used respectively the information on the disease-induced impairment, disability, threat to life, and overall unmet need as predictors in the regression models. The regression results obtained with the largest and smallest subsets can be found in Appendix G, including the standardized estimates. The standardized or beta coefficients represent how many standard deviations the relative importance will change for each standard deviation increase in the predictor variable. As difference scales were used to measure the context variables, only the standardized estimates can be used to check which variable has the largest effect on the value attached to each attribute.

We summarized the regression results in the manuscript in Figure 3 in terms of the size of the most significant effects observed. The result of the sensitivity analysis representing how often an effect was found significant can be found in Appendix G.
