# Supplementary material for: Quantifying benefit-risk preferences for new medicines in rare disease patients and caregivers
Source: Orphanet J Rare Dis. 2016 May 26;11:70. doi: 10.1186/s13023-016-0444-9 (PMC4881055; doi:10.1186/s13023-016-0444-9)

Appendix F – Study Sample

The following tables and graphs describe the main characteristics of our study sample. Characteristics of sub-groups are shown where appropriate. The (*) indicates the 10 most represented rare diseases in the study sample.

Table 1: Study participants, described by patient organisation and disease

| **Participating patient organisations and included rare conditions** | **Patients (n)** | **Caregivers (n)** | **Total sample, n (%)** |
| --- | --- | --- | --- |
| **Sarcoidosis & Interstitial Lung Association** | **404** | **19** | **423 (48.5)** |
| Sarcoidosis* | 401 | 17 | 418 |
| Other | 3 | 2 | 5 |
| **MPS Society - The Society for Mucopolysaccharide Diseases** | **53** | **47** | **100 (11.5)** |
| Fabry disease* | 32 | 4 | 36 |
| Fucosidosis |  | 4 | 4 |
| Mannosidosis |  | 2 | 2 |
| ML III - Pseudo Hurler Polydystrophy | 1 |  | 1 |
| MPS1 - Hurler. Hurler Scheie. Scheie* | 8 | 8 | 16 |
| MPS2 - Hunter disease | 3 | 6 | 9 |
| MPS3 - Sanfilippo disease |  | 11 | 11 |
| MPS4 - Morquio disease | 7 | 9 | 16 |
| MPS6 - Maroteaux-Lamy disease | 2 | 3 | 5 |
| **The Pituitary Foundation** | **48** | **7** | **55 (6.3)** |
| Acromegaly | 5 |  | 5 |
| Cushing's disease | 6 | 1 | 7 |
| Growth hormone deficiency | 1 |  | 1 |
| Hypopituitarism* | 23 | 3 | 26 |
| Prolactinoma | 7 |  | 7 |
| Septo-optic dysplasia |  | 2 | 2 |
| Sheehan's syndrome | 1 |  | 1 |
| Other | 5 | 1 | 6 |
| **MND Association - Motor Neurone Disease Association** | **34** | **19** | **53 (6.1)** |
| Amyotrophic lateral sclerosis - Charcot disease* | 13 | 11 | 24 |
| Kennedy's disease | 1 | 1 | 2 |
| Primary lateral sclerosis | 8 |  | 8 |
| Progressive bulbar palsy | 6 | 7 | 13 |
| Progressive muscular atrophy | 3 |  | 3 |
| Other | 3 |  | 3 |
| **AGSD (UK) - The Association for Glycogen Storage Disease** | **34** | **12** | **46 (5.3)** |
| Cori disease - Glycogen storage disease type 3 |  | 2 | 2 |
| Glycogen storage disease type 9 | 2 |  | 2 |
| Hers disease - Glycogen storage disease type 6 | 1 | 1 | 2 |
| McArdle's disease - Glycogen storage disease type 5* | 15 | 1 | 16 |
| Pompe disease - Glycogen storage disease type 2* | 14 | 5 | 19 |
| Tarui disease - Glycogen storage disease type 7 |  | 1 | 1 |
| Von Gierke disease - Glycogen storage disease type 1 | 1 | 2 | 3 |
| Other | 1 |  | 1 |
| **Ataxia UK** | **30** | **4** | **34 (3.9)** |
| Ataxia with vitamin E deficiency | 2 |  | 2 |
| Episodic ataxia | 1 |  | 1 |
| Friedreich ataxia | 4 | 1 | 5 |
| Spinocerebellar ataxia type 1 | 1 |  | 1 |
| Spinocerebellar ataxia type 2 | 1 |  | 1 |
| Spinocerebellar ataxia type 3 | 2 |  | 2 |
| Spinocerebellar ataxia type 6 | 4 |  | 4 |
| Spinocerebellar ataxia type 17 | 1 |  | 1 |
| Other | 14 | 3 | 17 |
| **Transverse Myelitis Society** | **33** | **1** | **34 (3.9)** |
| Recurrent transverse myelitis | 6 |  | 6 |
| Transverse myelitis* | 25 | 1 | 26 |
| Other | 2 |  | 2 |
| **Brittle Bone Society** | **18** | **9** | **27 (3.1)** |
| Osteogenesis imperfecta - type 1 | 8 | 3 | 11 |
| Osteogenesis imperfecta - type 2 | 1 | 1 | 2 |
| Osteogenesis imperfecta - type 3 | 3 | 2 | 5 |
| Osteogenesis imperfecta - type 4 | 4 | 3 | 7 |
| Other | 2 |  | 2 |
| **Action Duchenne** | **4** | **22** | **26 (3.0)** |
| Duchenne muscular dystrophy* | 4 | 22 | 26 |
| **PKD Charity - The Polycystic Kidney Disease Charity** | **21** | **2** | **23 (2.6)** |
| Autosomal dominant polycystic kidney disease* | 17 | 1 | 18 |
| Autosomal recessive polycystic kidney disease | 2 | 1 | 3 |
| Other | 2 |  | 2 |
| **Sickle Cell Society** | **9** | **4** | **13 (1.5)** |
| Sickle cell anaemia | 9 | 4 | 13 |
| **AKU - Alkaptonuria Society** | **8** | **2** | **10 (1.1)** |
| Alkaptonuria (AKU) | 8 | 2 | 10 |
| **Behcet's Syndrome Society** | **10** |  | **10 (1.1)** |
| Behcet's syndrome | 10 |  | 10 |
| **Facioscapulohumeral Muscular Dystrophy Support Group UK** | **8** |  | **8 (0.9)** |
| Facioscapulohumeral muscular dystrophy 1A | 8 |  | 8 |
| **MDSG - Myotonic Dystrophy Support Group** | **4** | **4** | **8 (0.9)** |
| Myotonic dystrophy type 1 | 4 | 4 | 8 |
| **Myaware** | **3** |  | **3 (0.3)** |
| Myasthenia gravis | 3 |  | 3 |
| **Total sample** | **721** | **152** | **873 (100.0)** |

Table 2: Study participants, described by socio-demographic characteristics and disease

|  | **Patients** | **Caregivers** | **Total sample** |
| --- | --- | --- | --- |
| **All study participants** |  |  |  |
| **Study eligible participants, n (%)** | 721 (82.6) | 152 (17.4) | 873 (100.0) |
| **Gender, n (%)** |  |  |  |
| Female | 462 (64.1) | 127 (83.6) | 589 (67.5) |
| Male | 259 (35.9) | 25 (16.4) | 284 (32.5) |
| **Age, n (%)** |  |  |  |
| 18 to 24 years | 18 (2.5) | 7 (4.6) | 25 (2.9) |
| 25 to 34 years | 56 (7.8) | 27 (17.8) | 83 (9.5) |
| 35 to 44 years | 154 (21.4) | 38 (25.0) | 192 (22.0) |
| 45 to 54 years | 196 (27.2) | 46 (30.3) | 242 (27.7) |
| 55 to 64 years | 198 (27.5) | 28 (18.4) | 226 (25.9) |
| > 65 years | 99 (13.7) | 6 (3.9) | 105 (12.0) |
| **Group of disease, n (%)** |  |  |  |
| Rare systemic or rheumatologic diseases | 414 (57.4) | 19 (12.5) | 433 (49.6) |
| Rare neurologic diseases | 116 (16.1) | 50 (32.9) | 166 (19.0) |
| Inborn errors of metabolism | 95 (13.2) | 61 (40.1) | 156 (17.9) |
| Rare endocrine diseases | 48 (6.7) | 5 (3.3) | 53 (6.1) |
| Rare bone diseases | 18 (2.9) | 9 (5.9) | 27 (3.1) |
| Rare renal diseases | 21 (2.5) | 2 (1.3) | 23 (2.6) |
| Rare hematologic diseases | 9 (1.2) | 4 (2.6) | 13 (1.5) |
| Rare developmental defect during embryogenesis |  | 2 (1.3) | 2 (0.2) |
| **Race, n (%)** |  |  |  |
| Caucasian | 633 (87.8) | 134 (88.2) | 767 (87.9) |
| Asian | 21 (2.9) | 1 (0.7) | 23 (2.6) |
| Black | 16 (2.2) | 7 (4.6) | 22 (2.5) |
| Mixed race | 16 (2.2) | 2 (1.3) | 18 (2.1) |
| Undisclosed | 4 (0.6) | 2 (1.3) | 37 (4.2) |
| Other | 31 (4.3) | 6 (3.9) | 6 (0.7) |
| **Education, n (%)** |  |  |  |
| Postgraduate qualification or above | 151 (20.9) | 32 (21.1) | 183 (21.0) |
| University first degree | 134 (18.6) | 28 (18.4) | 162 (18.6) |
| Advanced level qualification | 236 (32.7) | 46 (30.3) | 282 (32.3) |
| Secondary school qualification | 152 (21.1) | 32 (21.1) | 184 (21.1) |
| Other | 48 (6.7) | 14 (9.2) | 62 (7.1) |
| **Employment, n (%)** |  |  |  |
| Employed full-time | 197 (27.3) | 40 (26.3) | 237 (27.1) |
| Employed part-time | 80 (11.1) | 32 (21.1) | 112 (12.8) |
| Self-employed | 63 (8.7) | 12 (7.9) | 75 (8.6) |
| Student | 14 (1.9) | 4 (2.6) | 18 (2.1) |
| Retired | 136 (18.9) | 12 (7.9) | 148 (17.0) |
| Unable to work due to disability or other health problem | 195 (27.0) | 11 (7.2) | 206 (23.6) |
| Unable to work as fully dedicated to provide informal care | 4 (0.6) | 31 (20.4) | 35 (4.0) |
| Unemployed and looking for work | 16 (2.2) | 2 (1.3) | 18 (2.1) |
| Unemployed and not looking for work | 7 (1.0) |  | 7 (0.8) |
| Other | 9 (1.2) | 8 (5.3) | 17 (1.9) |
| **Living status, n (%)** |  |  |  |
| Living alone | 118 (16.4) | 6 (3.9) | 124 (14.2) |
| Living with other adults and/or children | 603 (83.6) | 146 (96.1) | 749 (85.8) |
| **Income, n (%)** |  |  |  |
| < £20,000 | 177 (24.5) | 21 (13.8) | 198 (22.7) |
| £21,000 - £35,000 | 132 (18.3) | 20 (13.2) | 152 (17.4) |
| £36,000 - £50,000 | 110 (15.3) | 37 (24.3) | 147 (16.8) |
| £51,000 - £75,000 | 88 (12.2) | 18 (11.8) | 106 (12.1) |
| £76,000 - £99,000 | 32 (4.4) | 9 (5.9) | 41 (4.7) |
| > £100,000 | 46 (6.4) | 9 (5.9) | 55 (6.3) |
| Undisclosed | 136 (18.9) | 38 (25.0) | 174 (19.9) |
| **Religion, n (%)** |  |  |  |
| Buddhism | 5 (0.7) | 1 (0.7) | 6 (0.7) |
| Catholicism | 84 (11.7) | 21 (13.8) | 105 (12.0) |
| Christianity | 207 (28.7) | 46 (30.3) | 253 (29.0) |
| Hinduism | 8 (1.1) | 2 (1.3) | 10 (1.1) |
| Inter/Non-denominational | 10 (1.4) | 2 (1.3) | 12 (1.4) |
| Islam | 10 (1.4) | 6 (3.9) | 16 (1.8) |
| Judaism | 16 (2.2) |  | 16 (1.8) |
| Protestantism | 96 (13.3) | 12 (7.9) | 108 (12.4) |
| No religion | 254 (35.2) | 54 (35.5) | 308 (35.3) |
| Undisclosed | 31 (4.3) | 8 (5.3) | 39 (4.5) |
| **Sub-group descriptive analysis on treatment** |  |  |  |
| **Patient currently under treatment?, n (%)** |  |  |  |
| Yes | 510 (70.7) | 126 (82.9) | 636 (72.9) |
| No | 211 (29.3) | 26 (17.1) | 237 (27.1) |
| **Channel to receive treatment, n (%)** |  |  |  |
| Through the National Health Service | 446 (87.5) | 110 (87.3) | 556 (87.4) |
| Through private health insurance | 56 (11.0) | 5 (4.0) | 61 (9.6) |
| By participating to a clinical trial | 8 (1.6) | 8 (6.3) | 16 (2.5) |
| Through a compassionate use programme | 0 (0) | 3 (2.4) | 3 (0.5) |
| **Subgroup analysis on patients’ experience on care delivery** |  |  |  |
| **Time since diagnosis, n (%)** |  |  |  |
| Less than a year ago | 72 (10.0) |  |  |
| Between 2 - 5 years | 233 (32.3) |  |  |
| Between 6 - 10 years | 153 (21.2) |  |  |
| Between 11 - 15 years | 91 (12.6) |  |  |
| More than 16 years ago | 172 (23.9) |  |  |
| **Patients receiving informal care, n (%)** |  |  |  |
| Yes | 432 (59.9) |  |  |
| No | 289 (40.1) |  |  |
| **Subgroup analysis on caregivers’ experience on care delivery** |  |  |  |
| **Number of years as caregiver, n (%)** |  |  |  |
| < 1year |  | 19 (12.5) |  |
| Between 2 - 5 years |  | 41 (27.0) |  |
| Between 6 - 10 years |  | 34 (22.4) |  |
| Between 11 - 15 years |  | 21 (13.8) |  |
| Over 16 years |  | 37 (24.3) |  |
| **Number of hours per week dedicated to informal care, n (%)** |  |  |  |
| <10 hours |  | 33 (21.7) |  |
| Between 11 - 36 hours |  | 29 (19.1) |  |
| Between 37 - 60 hours |  | 23 (15.1) |  |
| Over 60 hours |  | 60 (39.5) |  |
| Most care is given by a care team |  | 7 (4.6) |  |
| **Type of Patient-to-Caregiver relationship, n (%)** |  |  |  |
| Parent or legal guardian |  | 97 (63.8) |  |
| Spouse |  | 28 (18.4) |  |
| Family member |  | 26 (17.1) |  |
| Friend |  | 1 (0.7) |  |
| **Caregiver to** **, n (%)** |  |  |  |
| a child |  | 74 (48.7) |  |
| an adult |  | 78 (51.3) |  |

Table 3: Self- or proxy-reported assessment on disease context, across the whole study sample

|  | **Patients (n: 721)** | **Caregivers (n:152)** | **Total sample (n: 873)** |
| --- | --- | --- | --- |
| **WHODAS 2.0 12-item simple sum mean score (SD), 0 - 48 scale** | 16.6 (11.7) | 26.2 (13.4) | 18.3 (12.5) |
| **Reported threat to life assessment, mean (SD)** | 4.8 (3.0) | 7.7 (2.8) | 5.3 (3.2) |
| **Reported disease-induced impairment assessment, mean (SD)** | 3.2 (2.2) | 5.1 (3.0) | 3.5 (2.5) |
|  |  |  |  |

Table 4: Self- or proxy-reported assessment on disease context across the sub-group for on-going treatment

|  | **Patients (n: 510)** | **Caregivers (n:126)** | **Total sample (n: 636)** |
| --- | --- | --- | --- |
| **Satisfaction with treatment, n (%)** |  |  |  |
| Very unsatisfied | 40 (7.8) | 16 (12.7) | 56 (8.8) |
| Unsatisfied | 96 (18.8) | 18 (14.3) | 114 (17.9) |
| Neutral | 129 (25.3) | 34 (27.0) | 163 (25.6) |
| Satisfied | 187 (36.7) | 41 (32.5) | 228 (35.8) |
| Very satisfied | 58 (11.4) | 17 (13.5) | 75 (11.8) |
| **Current disease management, n (%)** |  |  |  |
| Medical care does not bring any improvement in health condition | 52 (10.2) | 17 (13.5) | 69 (10.8) |
| Medical care moderately extends survival | 41 (8.0) | 12 (9.5) | 53 (8.3) |
| Medical care helps improve quality of life | 89 (17.5) | 29 (23.0) | 118 (18.6) |
| Medical care manages the main symptoms | 161 (31.6) | 30 (23.8) | 191 (30.0) |
| Medical care prevents health condition form getting worse | 113 (22.2) | 25 (19.8) | 138 (21.7) |
| Medical care improves health condition | 46 (9.0) | 11 (8.7) | 57 (9.0) |
| Getting cured | 8 (1.6) | 2 (1.6) | 10 (1.6) |
| **Impact of medical care on day-to-day life, n (%)** |  |  |  |
| No impact | 159 (31.2) | 27 (21.4) | 186 (29.2) |
| Some impact | 322 (63.1) | 76 (60.3) | 398 (62.6) |
| Most of the day dedicated to medical care | 29 (5.7) | 23 (18.3) | 52 (8.2) |

Graph 1: Distribution of scores on reported assessment on disease context


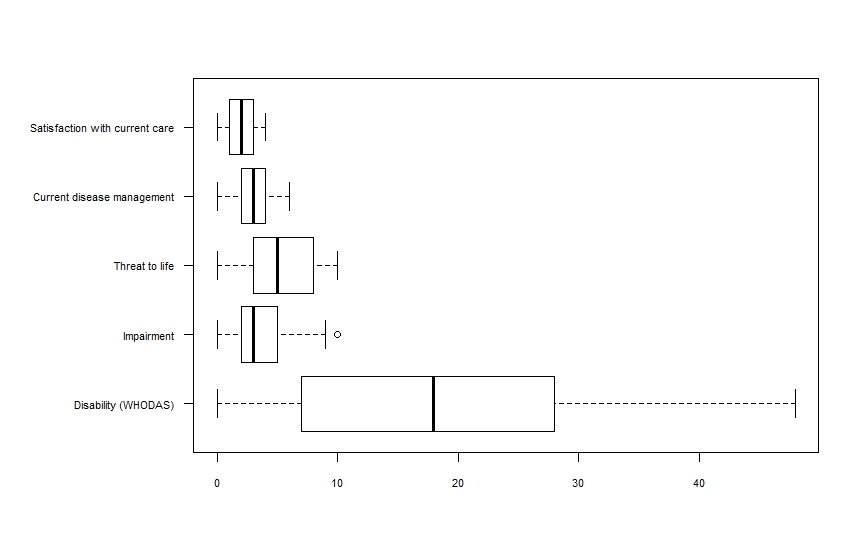


**Graph 2: WHODAS 2.0 scores described by respondent type**


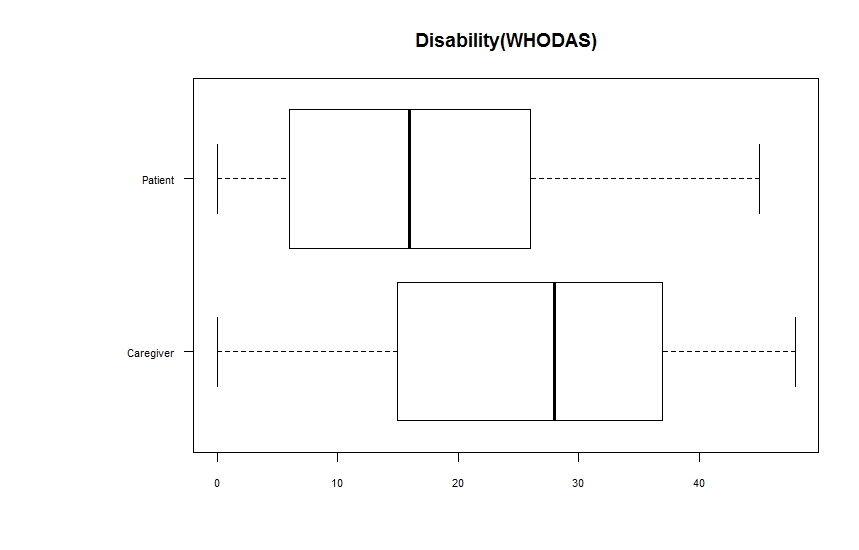

Supplement: Supplementary file 6 — Study Sample. (DOCX 133 kb) [file 13023_2016_444_MOESM6_ESM.docx]
