# Supplementary material for: Quantifying benefit-risk preferences for new medicines in rare disease patients and caregivers
Source: Orphanet J Rare Dis. 2016 May 26;11:70. doi: 10.1186/s13023-016-0444-9 (PMC4881055; doi:10.1186/s13023-016-0444-9)

Appendix G – Mixed logit estimates & regression output

## Mixed logit estimates

Some descriptive statistics on the individual part-worths as estimated on the dataset with choices of 873 respondents are given in the following table.

The results were obtained by hierarchical Bayes estimation using the R-package bayesm.

| **Attribute levels** | **Mean** | **Std. Dev.** | **Minimum** | **Maximum** | **1st Quartile** | **Median** | **3rd Quartile** |
| --- | --- | --- | --- | --- | --- | --- | --- |
| **alt1_a** | 1,9578 | 0,8053 | -0,6526 | 3,6029 | 1,4287 | 1,9831 | 2,5729 |
| **alt1_b** | 0,1885 | 0,4405 | -1,3690 | 1,3777 | -0,1107 | 0,2231 | 0,5193 |
| **alt1_c** | -2,1462 | 0,9745 | -4,1850 | 1,0069 | -2,9145 | -2,1716 | -1,4482 |
| **alt2_a** | 1,668 | 1,286 | -1,737 | 4,011 | 0,698 | 1,717 | 2,665 |
| **alt2_b** | -0,4014 | 0,3946 | -1,4807 | 0,8940 | -0,6570 | -0,4101 | -0,1483 |
| **alt2_c** | -1,267 | 1,234 | -4,005 | 1,978 | -2,158 | -1,301 | -0,372 |
| **alt3_a** | 0,9216 | 0,7281 | -1,3363 | 2,6576 | 0,3902 | 0,9900 | 1,4467 |
| **alt3_b** | 0,3239 | 0,4457 | -1,2007 | 1,7739 | 0,0549 | 0,3303 | 0,6291 |
| **alt3_c** | -1,2455 | 0,7286 | -2,9804 | 0,7472 | -1,7713 | -1,2962 | -0,7406 |
| **alt4_a** | 1,371 | 1,106 | -1,575 | 3,836 | 0,614 | 1,408 | 2,207 |
| **alt4_b** | 0,3959 | 0,5203 | -1,2101 | 1,7291 | 0,0796 | 0,3989 | 0,7400 |
| **alt4_c** | -1,767 | 1,225 | -4,634 | 1,577 | -2,679 | -1,794 | -0,918 |
| **alt5_a** | 0,6711 | 0,6413 | -1,2220 | 2,3300 | 0,2643 | 0,7084 | 1,0851 |
| **alt5_b** | 0,2733 | 0,3911 | -1,0894 | 1,5064 | 0,0043 | 0,2977 | 0,5164 |
| **alt5_c** | -0,9444 | 0,7664 | -2,8718 | 1,3272 | -1,4798 | -0,9546 | -0,4782 |
| **alt6_a** | 0,9217 | 0,6997 | -0,8818 | 2,7703 | 0,4308 | 0,8956 | 1,4175 |
| **alt6_b** | 0,0101 | 0,4601 | -1,4770 | 1,4061 | -0,2787 | 0,0067 | 0,3359 |
| **alt6_c** | -0,9319 | 0,6695 | -2,7478 | 0,8852 | -1,3729 | -0,9059 | -0,4463 |
| **alt7_a** | 1,3831 | 0,9525 | -1,5800 | 3,7343 | 0,6457 | 1,4077 | 2,1280 |
| **alt7_b** | 0,3838 | 0,5733 | -1,3657 | 1,7478 | -0,0005 | 0,3862 | 0,7932 |
| **alt7_c** | -1,767 | 1,147 | -4,148 | 1,375 | -2,684 | -1,796 | -0,835 |

## Regression output

|  |  |  |  |  |  |  |  |  |  |  |  |  |
| --- | --- | --- | --- | --- | --- | --- | --- | --- | --- | --- | --- | --- |
|  |  | **Regression outcomes (873 observations)** | | | | | **Regression outcomes (298 observations)*** | | | | | **# of datasets where significance  was found** |
| Dependent (Attribute) | Variable | Standardized estimate | Estimate | StdErr | tValue | Probt | Standardized estimate | Estimate | StdErr | tValue | Probt |  |
| Chance that the medicine will work | Current disease management | 0,12882 | 0,01011 | 0,00309 | 3,27 | 0,0011 | 0,09551 | 0,00170 | 0,00118 | 1,45 | 0,1488 | 6 |
| Chance that the medicine will work | Impairment | 0,05699 | 0,00276 | 0,00164 | 1,68 | 0,0924 | 0,15246 | 0,00175 | 0,00066 | 2,65 | 0,0084 | 8 |
| Chance that the medicine will work | Threat to life | 0,03960 | 0,00149 | 0,00127 | 1,17 | 0,2425 | 0,19435 | 0,00168 | 0,00049 | 3,41 | 0,0007 | 6 |
| Chance that the medicine will work | Satisfaction with current care | 0,04664 | 0,00471 | 0,00400 | 1,18 | 0,2402 | 0,04764 | 0,00113 | 0,00157 | 0,72 | 0,4722 | 0 |
| Chance that the medicine will work | Unmet need* | 0,11863 | 0,00587 | 0,00195 | 3,01 | 0,0027 | 0,11138 | 0,00133 | 0,00079 | 1,69 | 0,0919 | 1 |
| Chance that the medicine will work | Disability (WHODAS) | 0,04672 | 0,00044 | 0,00032 | 1,38 | 0,1678 | 0,16981 | 0,00039 | 0,00013 | 2,96 | 0,0033 | 8 |
| Expected health improvement | Current disease management | 0,05470 | 0,00342 | 0,00248 | 1,38 | 0,1683 | 0,03039 | 0,00121 | 0,00263 | 0,46 | 0,6466 | 0 |
| Expected health improvement | Impairment | 0,09838 | 0,00374 | 0,00128 | 2,92 | 0,0036 | 0,22953 | 0,00559 | 0,00138 | 4,06 | <,0001 | 9 |
| Expected health improvement | Threat to life | 0,07693 | 0,00228 | 0,00100 | 2,28 | 0,0230 | 0,13743 | 0,00251 | 0,00105 | 2,39 | 0,0176 | 9 |
| Expected health improvement | Satisfaction with current care | 0,09529 | 0,00765 | 0,00317 | 2,41 | 0,0162 | 0,07472 | 0,00396 | 0,00350 | 1,13 | 0,2591 | 7 |
| Expected health improvement | Unmet need* | 0,09184 | 0,00362 | 0,00156 | 2,32 | 0,0205 | 0,07328 | 0,00196 | 0,00176 | 1,11 | 0,2684 | 3 |
| Expected health improvement | Disability (WHODAS) | 0,09508 | 0,00071 | 0,00025 | 2,82 | 0,0049 | 0,20980 | 0,00102 | 0,00028 | 3,69 | 0,0003 | 9 |
| Risk of moderate side effects | Current disease management | 0,04271 | 0,00178 | 0,00165 | 1,08 | 0,2822 | 0,08423 | 0,00146 | 0,00114 | 1,28 | 0,2031 | 0 |
| Risk of moderate side effects | Impairment | 0,04712 | 0,00131 | 0,00094 | 1,39 | 0,1642 | -0,06025 | -0,00065 | 0,00063 | -1,04 | 0,2999 | 0 |
| Risk of moderate side effects | Threat to life | 0,08760 | 0,00189 | 0,00073 | 2,6 | 0,0096 | -0,01634 | -0,00013 | 0,00047 | -0,28 | 0,7788 | 2 |
| Risk of moderate side effects | Satisfaction with current care | -0,02441 | -0,00130 | 0,00212 | -0,61 | 0,5390 | 0,05425 | 0,00125 | 0,00153 | 0,82 | 0,4129 | 0 |
| Risk of moderate side effects | Unmet need* | 0,03009 | 0,00079 | 0,00104 | 0,76 | 0,4487 | 0,07679 | 0,00089 | 0,00077 | 1,16 | 0,2461 | 0 |
| Risk of moderate side effects | Disability (WHODAS) | 0,04525 | 0,00025 | 0,00018 | 1,34 | 0,1817 | -0,05116 | -0,00011 | 0,00013 | -0,88 | 0,3788 | 0 |
| Risk of serious side effects | Current disease management | -0,11248 | -0,00783 | 0,00275 | -2,85 | 0,0045 | -0,14866 | -0,00417 | 0,00184 | -2,27 | 0,0242 | 8 |
| Risk of serious side effects | Impairment | -0,06710 | -0,00284 | 0,00143 | -1,98 | 0,0475 | -0,18030 | -0,00314 | 0,00099 | -3,15 | 0,0018 | 9 |
| Risk of serious side effects | Threat to life | -0,02791 | -0,00092 | 0,00112 | -0,82 | 0,4102 | -0,22298 | -0,00291 | 0,00074 | -3,94 | 0,0001 | 7 |
| Risk of serious side effects | Satisfaction with current care | -0,04760 | -0,00426 | 0,00355 | -1,2 | 0,2307 | -0,08763 | -0,00328 | 0,00247 | -1,33 | 0,1854 | 0 |
| Risk of serious side effects | Unmet need* | -0,10557 | -0,00464 | 0,00173 | -2,67 | 0,0077 | -0,17210 | -0,00324 | 0,00123 | -2,64 | 0,0089 | 2 |
| Risk of serious side effects | Disability (WHODAS) | -0,09196 | -0,00076 | 0,00028 | -2,73 | 0,0065 | -0,15635 | -0,00054 | 0,00020 | -2,72 | 0,0068 | 9 |
| Treatment duration | Current disease management | 0,02008 | 0,00075 | 0,00148 | 0,51 | 0,6133 | -0,02472 | -0,00030 | 0,00081 | -0,37 | 0,7092 | 0 |
| Treatment duration | Impairment | -0,06095 | -0,00140 | 0,00078 | -1,8 | 0,0719 | 0,05025 | 0,00039 | 0,00045 | 0,87 | 0,3874 | 0 |
| Treatment duration | Threat to life | -0,04742 | -0,00085 | 0,00061 | -1,4 | 0,1615 | 0,11652 | 0,00068 | 0,00034 | 2,02 | 0,0444 | 2 |
| Treatment duration | Satisfaction with current care | 0,03159 | 0,00151 | 0,00190 | 0,8 | 0,4264 | -0,00532 | -0,00009 | 0,00108 | -0,08 | 0,9360 | 0 |
| Treatment duration | Unmet need* | 0,02714 | 0,00064 | 0,00093 | 0,68 | 0,4944 | -0,02741 | -0,00022 | 0,00054 | -0,41 | 0,6793 | 1 |
| Treatment duration | Disability (WHODAS) | -0,04282 | -0,00019 | 0,00015 | -1,26 | 0,2063 | -0,01168 | -0,00002 | 0,00009 | -0,2 | 0,8408 | 1 |
| Burden of treatment | Current disease management | -0,03061 | -0,00188 | 0,00243 | -0,77 | 0,4409 | 0,04799 | 0,00080 | 0,00111 | 0,73 | 0,4690 | 0 |
| Burden of treatment | Impairment | -0,00570 | -0,00022 | 0,00130 | -0,17 | 0,8664 | -0,14485 | -0,00152 | 0,00060 | -2,52 | 0,0123 | 6 |
| Burden of treatment | Threat to life | -0,09129 | -0,00273 | 0,00101 | -2,71 | 0,0070 | -0,07587 | -0,00060 | 0,00046 | -1,31 | 0,1915 | 4 |
| Burden of treatment | Satisfaction with current care | -0,00805 | -0,00064 | 0,00313 | -0,2 | 0,8394 | 0,00530 | 0,00012 | 0,00148 | 0,08 | 0,9363 | 0 |
| Burden of treatment | Unmet need* | -0,03800 | -0,00147 | 0,00154 | -0,96 | 0,3387 | 0,04121 | 0,00046 | 0,00075 | 0,62 | 0,5341 | 1 |
| Burden of treatment | Disability (WHODAS) | -0,00288 | -0,00002 | 0,00026 | -0,09 | 0,9322 | -0,13179 | -0,00028 | 0,00012 | -2,29 | 0,0229 | 6 |
| Ability to conduct usual activities while on treatment | Current disease management | -0,09922 | -0,00634 | 0,00252 | -2,51 | 0,0123 | -0,03209 | -0,00071 | 0,00146 | -0,48 | 0,6283 | 1 |
| Ability to conduct usual activities while on treatment | Impairment | -0,08574 | -0,00335 | 0,00132 | -2,54 | 0,0113 | -0,17435 | -0,00242 | 0,00080 | -3,05 | 0,0025 | 8 |
| Ability to conduct usual activities while on treatment | Threat to life | -0,03816 | -0,00116 | 0,00103 | -1,13 | 0,2600 | -0,11740 | -0,00122 | 0,00060 | -2,03 | 0,0428 | 1 |
| Ability to conduct usual activities while on treatment | Satisfaction with current care | -0,09339 | -0,00767 | 0,00325 | -2,36 | 0,0185 | -0,10542 | -0,00311 | 0,00194 | -1,6 | 0,1108 | 4 |
| Ability to conduct usual activities while on treatment | Unmet need* | -0,11937 | -0,00481 | 0,00159 | -3,03 | 0,0026 | -0,07989 | -0,00119 | 0,00098 | -1,21 | 0,2275 | 7 |
| Ability to conduct usual activities while on treatment | Disability (WHODAS) | -0,05497 | -0,00042 | 0,00026 | -1,62 | 0,1046 | -0,16655 | -0,00046 | 0,00016 | -2,91 | 0,0039 | 7 |
| * Results not reported in manuscript |  |  |  |  |  |  |  |  |  |  |  |  |

## Regression output, sensitivity analysis


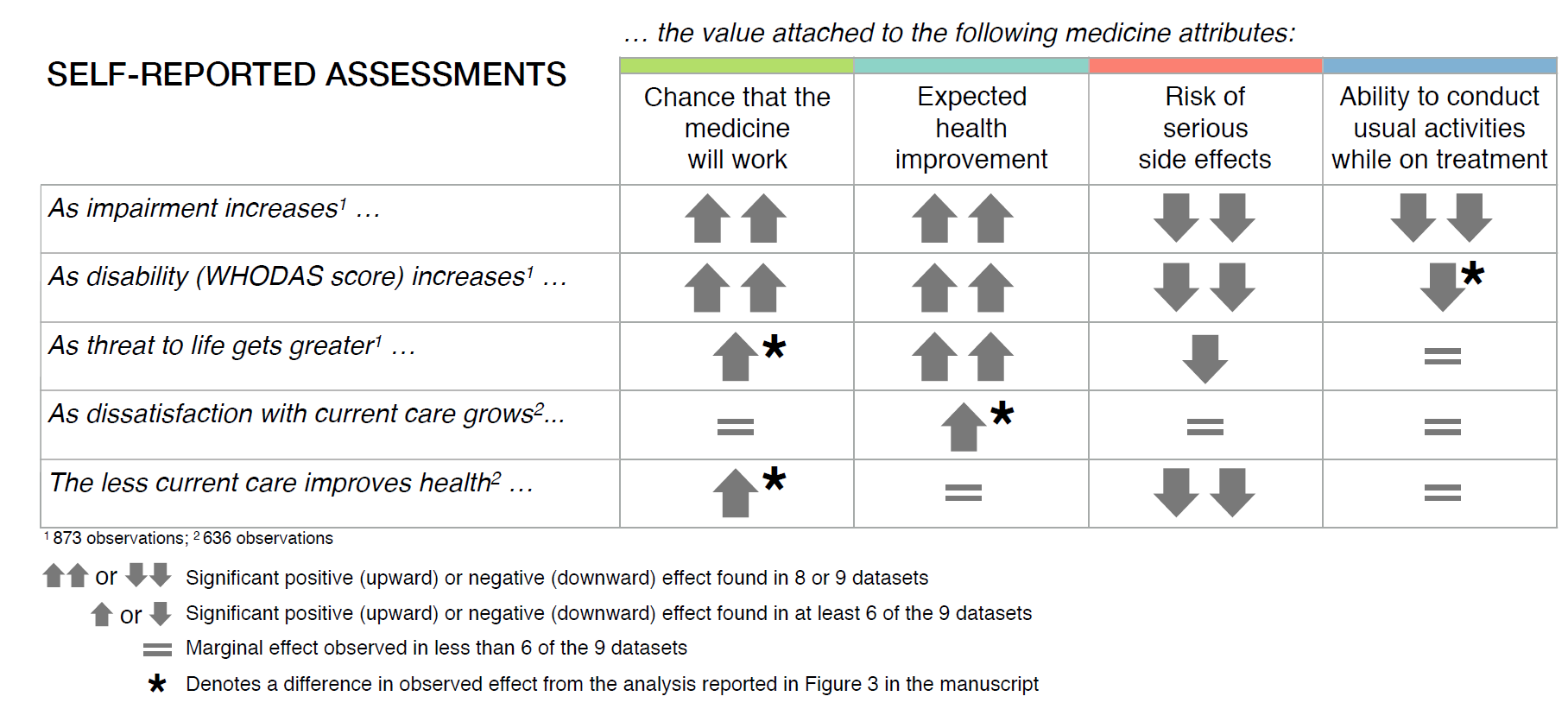

Supplement: Supplementary file 7 — Mixed logit estimates & regression output. (DOCX 186 kb) [file 13023_2016_444_MOESM7_ESM.docx]
