# Supplementary material for: Quantifying benefit-risk preferences for new medicines in rare disease patients and caregivers
Source: Orphanet J Rare Dis. 2016 May 26;11:70. doi: 10.1186/s13023-016-0444-9 (PMC4881055; doi:10.1186/s13023-016-0444-9)
Supplement: Supplementary file 8 — Patients’ quotations on ‘the value of a medicine’. (DOCX 25 kb) [file 13023_2016_444_MOESM8_ESM.docx]

Appendix H – Patients’ comments on ‘the value of a medicine’

**HOPE & PRAGMATISM**

1. *‘I will try any medicine that will give hope of recovery or partial recovery’*
2. *‘I have always been hopeful for something to make a difference to my condition’*
3. *‘****To have no hope is to have no life or anything to live for*** *– any chance that any research brings of not just a cure but hope that their life will be longer or will not deteriorate makes a difference beyond anything’*
4. *‘Drinking Aloe Vera really helps my condition, it has enabled me to walk’*
5. *‘****We live in hope’***
6. *‘****Any port in the storm’***
7. *‘I chose to take less risk of dying as a result of taking the medication because I have maybe years for better treatments to arrive’*
8. *‘Any new medicine that can give some hope to those suffering with such diseases is invaluable. Currently, those suffering are living under a* ***traumatic and almost indescribable death sentence****’*
9. *‘I would prefer research into new medicines that will help with my bone, joint, muscle pain and tiredness, which are my main problems,* ***rather than research into a 'cure'*** *for my condition’*

**ATTITUDE TO RISK**

1. *‘My life has been badly affected by this condition.* ***Trying new medicines are worth the risk*** *in my view’*
2. *‘I have a damned if you do, damned if you don't mentality when it comes to not treating AKU versus taking medications with some risk involved. If the treatment shows great success in treating AKU but involves some risk I am open to trying it’*
3. *‘When you are extremely ill and have a life expectancy of less than 12 months, you are less conservative in your medicine choices and think nothing of side effects of pain and possible heart issues’*
4. *‘With Motor neurone disease any improvement outweighs any side effects.* ***Better to die than live with this cruel disease’***
5. *‘Because the condition is progressive,* ***I would consider any treatment****’*
6. *‘New medicines that offer hope to stop or stop and reverse symptoms are worth the risk of trying.* ***What's the worst that could happen, you die of MND no matter what at the moment****. If stem cell treatment was available with a small chance of stopping the disease but a high risk of side effects then we would take the chance. We have nothing to lose but everything to gain’*
7. *‘As I am symptom-free at present, I am against health risks. However,* ***once my condition deteriorates, my attitude to health risks will change****’*
8. *‘The side effects should not be underestimated or played down. You tend to go for treatment because the illness is the known thing you need help with, but the side effects are unknown so you take the risk. But ‘****minor’ side effects can be extremely wearing*** ***and challenging*** *when they occur every day. mental side effects are very difficult to manage’*
9. *‘Anything that can start to alleviate any of the condition or to cure it would be my preferred options & the risks are something I am willing to take to achieve this’*
10. *‘At the beginning of being ill I couldn't breathe, walk up stairs or look after my son and high doses of steroids got it under control and back on my feet so I would have taken anything at that time to function, however as the disease appeared to be lessening I would have been and was less inclined to want to take drugs with severe side effects’*
11. *Would have liked [to see in the survey] a "****risk not taking anything****" option!*
12. *‘I have discovered I am* ***better off with no medicine****, the side effects made me feel worse’*
13. *‘****Ultimately if I could live disease free, without impairment, I would risk anything*** *and do the hospital stays needed to just be able to function as a person, wife, mother, friend and daughter again’*

**CONCERNS OVER LONG-TERM STEROID TREATMENT**

1. *‘I take a range of medications, but the one I object to most is the steroid because of the physical changes it has made to me. This side effect may not be as relevant/important to other patients. This is* ***an area the medical profession need to be more sympathetic towards*** *as I believe it can strongly affect how/when a medication is accepted by a patient’*
2. *‘Doctors prescribe prednisone for Sarcoidosis and do not explain the side effects and long term effects and risks* ***without discussing it with you or giving you the options****. They got me into remission after 2 years but I am still suffering with other diseases caused by the Predinisone. I would have opted for other options if I was given a choice’*
3. *‘I look forward to a drug that does not have the side effects associated with steroids!!’*
4. *‘Whilst one wishes to be "cured" of an illness or condition, often the price to pay is considerable. I lead a normal life with my condition but should the point come when my consultant considers I need treatment, I know that* ***I would decline treatment with steroids or anti-cancer drugs*** *as I am very well aware of their side effects which have affected several people I know’*

**RELATIONSHIP WITH DOCTORS**

1. *‘Medications that are administered at hospital / clinic may seem less convenient than self-administered medications, but they bring* ***you in regular contact with health care professionals, which, in my case, is invaluable****’*
2. *‘****Doctors need to give us the information so we can decide for ourselves****, every medicine I have been given I didn't get any information other than the leaflet in the box. Overall consultants don't care about patients, they just want to reduce the queues’*
3. *‘****Anything that engages the sufferer in discussions determining how to handle treatment****/medication can only be beneficial to the patient’*
4. *‘I feel that health professionals take a very 'paternalistic' stance and give minimum information - "****they know best****"’*
5. *‘The worst is the lack of knowledge & understanding on all this -* ***total lack of understanding of the disease by most of the medical professionals*** *(they mostly won't even be told their thinking is from 50yrs past and* ***all the latest info is ready available – usually found by patients*** *long standing with the disease who have been through all the hoops and had to research their disease themselves, but this is* ***generally dismissed****/patients brushed off without looking into it!’*
6. *‘Too often very toxic treatments are offered on a 'let's see how this goes' basis seemingly without much consideration to efficacy and whether or not the benefits outweigh the side-effects. It makes the patient feel like they don't really matter and that* ***a form of Russian roulette is being played****. That, in addition to misdiagnoses indicate that the* ***medical profession need more training/education on certain illnesses****.*
7. *‘It would be insightful if doctors liaised with lung specialists to fully understand no two patients side effects are the same instead off the raised eyebrow & "are you sure".* ***Each new medicine should have the side effects explained in percentages like this survey****’*

**PATIENTS’ INTROSPECTION**

1. *‘Interesting survey that brought into focus my own evaluations of risk.* ***I had not really thought about it in exactly this way before****.’*
2. *‘very interesting survey making you focus on what really matters to you.’*
3. *‘Really interesting survey,* ***I learnt a lot about my attitude to risk****.’*
4. *‘I found the survey quite* ***thought provoking and made me think about my current treatment plan*** *and other options facing me.’*
5. *‘Answering the questions about value* ***made me realise*** *I regard the chance of a beneficial effect as outweighing any possible risk or discomfort.’*
