# Supplementary material for: Quantifying benefit-risk preferences for new medicines in rare disease patients and caregivers
Source: Orphanet J Rare Dis. 2016 May 26;11:70. doi: 10.1186/s13023-016-0444-9 (PMC4881055; doi:10.1186/s13023-016-0444-9)
Supplement: Supplementary file 9 — References used in supplementary material. (DOCX 21 kb) [file 13023_2016_444_MOESM9_ESM.docx]

Appendix I – References used in supplementary material

1. Andrews G, Kemp A, Sunderland M, von KM, Ustun TB: Normative data for the 12 item WHO Disability Assessment Schedule 2.0. *PLoS One* 2009, 4: e8343.

2. Kim JI, Long JD, Mills JA, Downing N, Williams JK, Paulsen JS: Performance of the 12-item WHODAS 2.0 in prodromal Huntington disease. *Eur J Hum Genet* 2015, 23: 1584-1587.

3. Carlozzi NE, Kratz AL, Downing NR, Goodnight S, Miner JA, Migliore N *et al*.: Validity of the 12-item World Health Organization Disability Assessment Schedule 2.0 (WHODAS 2.0) in individuals with Huntington disease (HD). *Qual Life Res* 2015, 24: 1963-1971.

4. Schlote A, Richter M, Wunderlich MT, Poppendick U, Moller C, Schwelm K *et al*.: WHODAS II with people after stroke and their relatives. *Disabil Rehabil* 2009, 31: 855-864.

5. Garin O, Ayuso-Mateos JL, Almansa J, Nieto M, Chatterji S, Vilagut G *et al*.: Validation of the "World Health Organization Disability Assessment Schedule, WHODAS-2" in patients with chronic diseases. *Health Qual Life Outcomes* 2010, 8: 51.

6. Bastiaens L, Galus J, Goodlin M: The 12 item W.H.O.D.A.S. as primary self report outcome measure in a correctional community treatment center for dually diagnosed patients. *Psychiatr Q* 2015, 86: 219-224.

7. Yu J, Goos P, Vandebroek M.: Efficient conjoint choice designs in the presence of respondent heterogeneity. *Marketing Science* 2009, 28: 122-135.

8. Yu J., Goos P, Vandebroek M.: Individually adapted sequential Bayesian conjoint-choice designs in the presence of consumer heterogeneity. *International Journal of Research in Marketing* 2011, 28: 378-388.

9. Yu J., Goos P, Vandebroek M.: A comparison of different Bayesian design criteria for setting up stated preference studies. *Transportation Research Part B* 2012, 46: 789-807.

10. Orme J: *Getting started with conjoint analysis: strategies for product design and pricing research*. Madison, USA: Research Publishers LLC; 2006.

11. Bliemer MCJ, Rose JM: Construction of Experimental Designs for Mixed Logit Models Allowing for Correlation Across Choice Observations. *Transportation Research Part B* 2010, 44: 720-734.
